# Alternative Ways to Obtain Human Mesenchymal Stem Cells from Embryonic Stem Cells

**DOI:** 10.3390/cells13191617

**Published:** 2024-09-26

**Authors:** Nikita Onyanov, Olga Glazova, Nawar Sakr, Tatyana Krokunova, Julia Krupinova, Pavel Volchkov

**Affiliations:** 1 Moscow Center for Advanced Studies, Kulakova Str. 20, Moscow 123592, Russia; 2 Federal Research Center for Innovator and Emerging Biomedical and Pharmaceutical Technologies, Moscow 125315, Russia; 3 Faculty of Fundamental Medicine, Lomonosov Moscow State University, Moscow 119992, Russia; 4 Moscow Clinical Scientific Center N.A. A.S. Loginov, Moscow 111123, Russia

**Keywords:** stem cell, differentiation, mesoderm, MSCs

## Abstract

Differentiation approaches to obtain mesenchymal stem cells (MSCs) have gradually developed over the last few decades. The problem is that different protocols give different MSC types, making further research difficult. Here, we tried three different approaches to differentiate embryonic stem cells (ESCs) from early mesoderm to MSCs using serum-containing or xeno-free differentiation medium and observed differences in the cells’ morphology, doubling rate, ability to form colonies, surface marker analysis, and multilineage differentiation potential of the obtained cell lines. We concluded that the xeno-free medium best fits the criteria of MSCs’ morphology, growth kinetics, and surface marker characterization. In contrast, the serum-containing medium gives better potential for further MSC differentiation into osteogenic, chondrogenic, and adipogenic lineages.

## 1. Introduction

Working with pluripotent stem cells (PSCs) is becoming increasingly popular for testing new cell-based methods, therapies, and research in regenerative medicine. PSCs can differentiate into the three germ layers: mesoderm, ectoderm, and endoderm.

Mesenchymal stem cells (MSCs) are multipotent stem cells that can self-renew and differentiate into bones, cartilage, tendon, ligament, marrow stroma, adipose tissue, dermis, muscle, and connective tissue [1,2]. These cells have fibroblast-like morphology [3], which may be slightly different depending on the cells’ origin [4].

MSCs, due to their embryonic development, are typically derivatives of early mesoderm cells [5]. It is possible to obtain them from bone marrow, adipose tissue, peripheral blood, dental pulp, and umbilical cord. However, the same cell types collected from different tissues and patients have different proliferation and differentiation potentials [6]. Understanding human embryonic development and differentiation pathways has made it possible to obtain MSCs from PSCs in different ways [7,8,9,10,11]. This approach may help obtain a universal standardized MSC line that can be used as a resource for differentiation to all other tissues.

This research compared three protocols for generating mesenchymal stem cells from embryonic stem cells to the early mesoderm stage. These results may be helpful for other research groups that are generating mesenchymal stem cells in their works and trying to use them for future differentiation experiments.

## 2. Materials and Methods

### 2.1. Cell Culture

Embryonic Stem Cells

Human embryonic stem cell line H9 was obtained from Dr. Samohvalov. It was cultured using mTesR1 (STEMCELL Technologies, Vancouver, BC, Canada, #85850). According to the manufacturer’s instructions, one well of an adhesive 6-well plate (SPL Life Sciences, Pocheon-si, Republic of Korea, #30006) was covered with Matrigel (Corning, NY, USA, #356231). Then, 100,000 cells in 2 mL of mTesR1 with 10 mkM Y-27632 (STEMCELL Technologies, Vancouver, BC, Canada, #72302) were plated on the well and incubated at +37 °C, 5% CO_2_, with daily medium change till 80–90% confluence. When confluent, all medium was aspirated from the cells. Then, the well was washed with 2 mL of DPBS (Gibco, Thermo Fisher Scientific, Waltham, MA, USA, #14040133). After that, 1 mL of Versen solution was added (Paneco, Moscow, Russia, P080п) and incubated at +37 °C, 5% CO_2_, for 5 min. After that, the Versen solution was removed, and the cells were washed from the well using 1 mL of mTesR1 with 10 mkM Y-27632. Then, 0.1 mL of the cell suspension was added to preheated mTesR1 (1.9 mL) with 10 mkM Y-27632 per well pre-covered with Matrigel, and incubated at +37 °C, 5% CO_2_ with daily medium change till 80–90% confluence.

### 2.2. MSCs.Control 

MSCs.Control were cultured using the MesenCult™-ACF Plus Culture Kit (STEMCELL Technologies, Vancouver, BC, Canada, #05448). One well of a 6-well plate was covered with 1 mL of the culture diluted 1:300 in DPBS (Gibco, Thermo Fisher Scientific, USA, #14040133) Animal Component-Free Cell attachment substrate (STEMCELL Technologies, Vancouver, BC, Canada, #07130) and incubated at room temperature for 2 h. Then, 80,000–100,000 cells were plated in 2 mL of Complete MesenCult™-ACF Plus medium on the well and incubated at +37 °C, 5% CO_2_ for 3–4 days till 80–90% confluence. Cell passage was conducted using an Animal Component-Free Cell Dissociation Kit (STEMCELL Technologies, Vancouver, BC, Canada, #05426) according to the manufacturer’s instructions.

### 2.3. MSCs.V1

MSCs.V1 were cultured using FBS-containing MesenCult™ Proliferation Kit (Human) (STEMCELL Technologies, Vancouver, BC, Canada, #05411). One well of a 6-well plate was covered with 0.1% gelatin (Paneco, Moscow, Russia, #Φ061) and incubated at room temperature for 30 min. After that, 0.1% gelatin was aspirated from the well; 80,000–100,000 cells were plated in 2 mL of Complete MesenCult™ medium and incubated at +37 °C, 5% CO_2_ for 5–7 days till 80–90% confluence with daily half-medium change. When cells reached confluence, all medium was aspirated from the well. Then, it was washed two times with 1 mL of DPBS and 0.5 mL of 0.25% Trypsin (Paneco, Moscow, Russia, #П036п) was added and incubated at +37 °C, 5% CO_2_, for 5 min. Then, 0.5 mL of Complete MesenCult™ medium was added to the well. Next, cells were washed from the well and moved to Corning® 15 mL centrifuge tubes (Corning, USA, #430791) and centrifuged at 300× *g* for 8 min with break-up. After that, all supernatant was removed and cells were resuspended with 1 mL of Complete MesenCult™ medium. Next, 0.33 mL of that solution was added to 1.7 mL of Complete MesenCult™ medium on a new well, pre-covered with 0.1% gelatin, of a six-well plate and incubated at +37 °C, 5% CO_2_ till 80–90% confluence.

### 2.4. MSCs.V2

MSCs.V2 were cultured using xeno-free chemically defined CellCor™ CD MSC medium (Xcell Therapeutics, Seoul, Republic of Korea, # YSP002). One well of a 6-well plate was covered with 0.1% gelatin and incubated at room temperature for 30 min. After that, 0.1% gelatin was aspirated from the well; 80,000–100,000 cells were plated in 2 mL of CellCor™ CD MSC medium and incubated at +37 °C, 5% CO_2_, for 3–4 days till 80–90% confluence. When cells reached confluence, all medium was aspirated from the well. Then, it was washed two times with 1 mL of DPBS and 0.5 mL of 0.25% Trypsin (Paneco, Moscow, Russia, #П036п) was added, and the cells were incubated at +37 °C, 5% CO_2_, for 5 min. Then, 0.5 mL of CellCor™ CD MSC medium was added to the well. Next, cells were washed from the well and moved to 9 mL of CellCor™ CD MSC medium in Corning® 15 mL centrifuge tubes (Corning, USA, #430791) and centrifuged at 300× *g* for 8 min with break-up. After that, all supernatant was removed and cells were resuspended with 1 mL of CellCor™ CD MSC medium. Next, cells were counted using an automated cell counter with trypan blue. After that, 80,000–100,000 cells were plated to the pre-covered well as described above.

## 3. Differentiation

### 3.1. Early Mesoderm Induction

For early mesoderm lineage induction, 500,000 H9 ESCs were seeded in one well of a 6-well plate pre-coated in Matrigel in 2 mL of mTesR1 medium, with 10 mkM Y-27632, and incubated for 24 h at +37 °C, 5% CO_2_. The next day, the medium was aspirated, the well washed once with 1 mL of DPBS, and 3 mL of STEMdiff™ Mesoderm Induction Medium (STEMCELL Technologies, Vancouver, BC, Canada, #05220) was added. This was incubated at +37 °C, 5% CO_2_ with daily 3 mL medium changes for 3 days. On day 5, the cells were ready for future differentiation and analysis. On the last day of mesoderm induction, all medium was aspirated and the well of the 6-well plate was washed with 1 mL of PBS. After that, the cells were fixed with 1 mL of 4% PFA and incubated at room temperature for 15 min. Then, the PFA was removed and the well was washed with 1 mL of PBS. After that, 1 mL of 0.2% TritonX100 (Bio-Rad Laboratories, Hercules, CA, USA, #1610407) was added and incubated at room temperature for 10 min. Then, the TritonX100 was aspirated and 1 mL of blocking buffer (2.5% BSA in PBS) was added and incubated on the shaker at room temperature for 30 min. Then, the blocking buffer was aspirated and 0.5 mL of diluted primary anti-Brachyury antibodies were added (ABclonal, Düsseldorf, Germany, #A16887) and incubated for 2 h at room temperature. After that, the well was washed three times with 0.1% Tween20 (Bio-Rad Laboratories, USA, #1706531) and was put on the shaker for 5 min. Then, 0.5 mL of diluted secondary anti-rabbit antibodies was added to the well and incubated for 30 min at room temperature, in the dark. Next, the well was washed two times with 0.1% Tween20, then placed on the shaker for 5 min. After that, 0.5 mL of diluted DAPI (Lumiprobe, Moscow, Russia, #1996) was added and incubated for 5 min at room temperature in the dark. Finally, the well was washed with 1 mL of PBS and 2 mL of PBS was added for future examination under fluorescent microscopy (EVOS3000M, ThermoFisher, USA).

### 3.2. Derivation of Mesenchymal Stem Cells 

On day 5, the early mesoderm induction medium was changed to 2 mL of Complete MesenCult™-ACF Plus medium, 2 mL of Complete MesenCult™ medium, or 2 mL of CellCor™ CD MSC medium (Xcell Therapeutics, Seoul, Republic of Korea, # YSP002) to obtain MSCs.Control, MSCs.V1, or MSCs.V2, respectively. After 24 h of incubation at +37 °C, 5% CO_2_, 2 mL of each medium was refreshed. The next day, 1 well of a 6-well plate was pre-coated with 1 mL of Animal Component-Free Cell attachment substrate (STEMCELL Technologies, Vancouver, BC, Canada, #07130) diluted 1:300 in DPBS (Gibco, USA, #14040133) for derivation of MSCs.Control, and 1 mL of 0.1% gelation solution for derivation of MSCs.V1 and MSCs.V2. Pre-coating was performed at room temperature for 2 h. After that, each well with cells was washed with 1 mL of DPBS. For MSCs.Control, cells were treated with the Animal Component-Free Cell Dissociation Kit (STEMCELL Technologies, Vancouver, BC, Canada, #05426), according to the manufacturer’s instructions. For MSCs.V1 and MSCs.V2, cells were treated with 0.5 mL of 0.25% Trypsin for 5 min at +37 °C, 5% CO_2_. Then, 2.5 mL of corresponding medium was added and the cell suspension was moved to 15 mL tubes, which were centrifuged for 8 min at 350× *g*, with break-up at room temperature. Afterward, in each 15 mL tube, supernatant was removed and each cell type was resuspended in 1 mL of the corresponding medium preheated to room temperature. After counting, 100,000 cells were added to 2 mL of the corresponding medium preheated to room temperature to each well of a 6-well plate, which was pre-covered in a corresponding way. For the next 2 weeks, cells were incubated at +37 °C, 5% CO_2_, with medium change every 3–4 days (MSCs.Control and MSCs.V2) and daily half-medium change (MSCs.V1).

The surface marker analysis was performed with the BD Stemflow™ Human MSC Analysis Kit (BD Biosciences, #562245) following the manufacturer’s instructions.

### 3.3. Osteogenic Differentiation

Osteogenic differentiation was performed with the MesenCult™ Osteogenic Differentiation Kit (Human) (STEMCELL Technologies, Vancouver, BC, Canada, #05465), following the manufacturer’s instructions. On day 21 of differentiation, all medium was aspirated and the well of the 6-well plate was washed with 1 mL of PBS. After that, cells were fixed with 1 mL of 4% PFA and incubated at room temperature for 15 min. Then, the PFA was removed and the well was washed with 1 mL of PBS. After that, cells were washed twice with 2 mL of PBS. Next, 1 mL of 1% Alizarin Red solution (Servicevio, Wuhan, Hubei, China, #G1038) was added. After 1 h of incubation at room temperature, cells were washed 3 times with 2 mL of running water. Next, 2 mL of running water was added for future examination under the Leica DM2000 microscopy system (Leica Microsystems, Wetzlar, Germany).

### 3.4. Chondrogenic Differentiation

Chondrogenic differentiation was performed with the MesenCult™ Chondrogenic Differentiation Kit (Human) (STEMCELL Technologies, Vancouver, Canada, #05455) following the manufacturer’s instructions. On day 21 of differentiation, all medium was aspirated and the obtained cell culture was washed with 1 mL of PBS. After that, cells were fixed with 1 mL of 4% PFA and incubated at +4 °C for 24 h. Then, the PFA was removed and the cells were washed twice with 1 mL of PBS. Next, 1 mL of 30% sucrose solution was added and incubated at +4 °C for 24 h. After that, the cells were washed twice with 1 mL of PBS, 1 mL of 15% sucrose solution was added, and they were incubated for 24 h at +4 °C. Next, the sucrose solution was removed, the cells were washed once with 1 mL of PBS, and the obtained cell structure was moved into a plastic form filled with embedding medium (Tissue-Tek ® 24 O.C.T. ™ Compound, Sakura Finetek, Torrance, CA, USA). The form was moved to a metallic block pre-frozen in liquid nitrogen and incubated till samples were fully frozen. The frozen samples were cut in 20 mkm slices using CryoTome FSE (ThermoScientific, USA) and were stuck onto poly-D-lysin-covered slides. After that, the slides were washed in running water and were stained in Harris Hematoxylin (DIAPATH, Bergamo, Italy, #C0283) for 5 min. Then, they were washed in 1% acetic acid and blued in ammonium water for 30 s. Next, the slides were rinsed in running water for 1 min and stained in Fast Green solution (Servicebio, Hubei, China, #G1051-1) for 5 min. After that, the slides were rinsed in running water for 30 s, incubated in 1% acetic acid for 30 s, then incubated in Safranin O solution (Servicebio, Hubei, China, #G1051-1). Subsequently, the slides were rinsed in running water for 30 s. Then, 4 more rounds of dehydration in 95% (round 1) and 100% (rounds 2, 3, and 4) were performed. After that, the sample was cleaned for 2 rounds in xylene, and the slides were coverslipped for future examination under the Leica DM2000 microscopy system.

### 3.5. Adipogenic Differentiation

Adipogenic differentiation was performed with the MesenCult™ Adipogenic Differentiation Kit (Human) (STEMCELL Technologies, Vancouver, BC, Canada, #05412) following the manufacturer’s instructions. On day 21 of differentiation, all medium was aspirated and the well of the 6-well plate was washed with 1 mL of PBS. After that, the cells were fixed with 1 mL of 4% PFA and incubated at room temperature for 15 min. Then, the PFA was removed and the well was washed with 1 mL of PBS. After that, the Oil Red O. working solution was prepared: 1 g of Oil Red O. (HermeticScience, Moscow, Russia) was diluted in 100 mL of isopropanol, 1 g of dextrin (Rushim, Moscow, Russia) was diluted in 100 mL of dH2O. These solutions were mixed 3:2 to obtain an Oil Red O. working solution. Next, 2 mL of this solution was added to the fixed cells and incubated for 10 min at room temperature. After that, the well was washed with 60% isopropanol 5 times. Next, the well was washed 3 times in running water. After that, 1 mL of Harris Hematoxylin (DIAPATH, Bergamo, Italy, #C0283) was added and incubated for 30 s. Finally, the well was washed 5 times with running water for future examination under the Leica DM2000 microscopy system.

## 4. Results

### 4.1. Existing Protocols for MSC Generation

Many published protocols for direct differentiation of PSCs to MSCs are based on standard laboratory reagents, such as FBS and 0.1% gelatine. To test if they can be used to obtain a well-established MSC culture, we first attempted to integrate various existing protocols for the one-step generation of mesenchymal stem cells (MSCs) from pluripotent stem cells (PSCs). We explored several matrices, including Matrigel, collagen, and 0.1% gelatin, to facilitate monolayer direct differentiation by seeding the cells on these substrates. To optimize the differentiation process, we used combinations of different basal media—DMEM/F12, DMEM-HG, and DMEM-LG—each supplemented with 10% fetal bovine serum. Additionally, these media were tested with and without the inclusion of sodium pyruvate, GlutaMax, or MEM Non-Essential Amino Acids (NEAAs) [12,13,14]. Unfortunately, all conditions yielded unsatisfactory results. The cells did not proliferate well and died by the end of the two-week direct differentiation attempts (data not shown). For this reason, we decided to test commercial kits and compare their ability to produce a strong MSC population.

### 4.2. Induction of Two-Step MSCs

**Figure 1:**
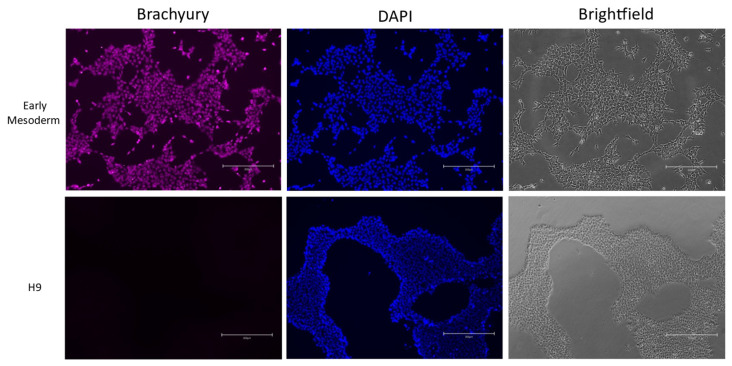
Confirmation of early mesoderm induction. Upper row—early mesoderm on day 4 of differentiation. Lower row—undifferentiated H9 ES cell line. First column—immunostaining with anti-Brachyury antibodies; second column—DAPI; third column—brightfield. Scale bar—300 mkm.

To better mimic normal embryonic development, we first differentiated cells into the early mesoderm stage. On the fifth day of monolayer differentiation (see Section 2), cells were stained to detect the presence of the main early mesoderm marker—Brachyury. We have demonstrated that ESCs do not express this marker at all, while most of the obtained cells express it (Figure 1). 

After confirmation of early mesoderm induction, the cells were differentiated into MSCs in three different ways. The MesenCult™-ACF Plus Culture Kit was used as a control because of its known reproducibility and efficiency. The MesenCult™ Proliferation Kit (Human) (MSCs.V1) was tested as a serum-containing medium by analogy with the direct differentiation protocols used at the beginning of our research.

CellCorTM CD medium (MSCs.V2) recently appeared on the market, a xeno-free complete medium that does not need any supplements, and is easier to use. 

**Figure 2:**
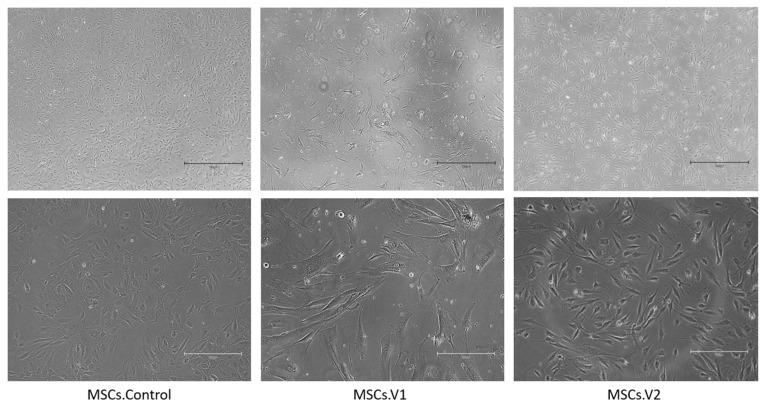
Differences in morphology of obtained cell lines. First column—MSCs.Control; second—MSCs.V1; third—MSCs.V2. Scale bar: upper row—750 mkm, lower row—300 mkm.

At day 21 of differentiation from PSCs (day 17 from early-mesoderm stage), the obtained cells had visually different morphology. The control’s differentiation to MSCs (Figure 2) and version 2’s differentiation yielded results similar to the expected fibroblast-like morphology. However, the third passage of version 1 MSCs displayed notable visual differences: the cells were significantly larger and did not appear as a homogeneous culture.

We then analyzed the cell doubling speed, although cells were not synchronized by cell cycle, which may contribute to larger error bars in the graphs. 

**Figure 3:**
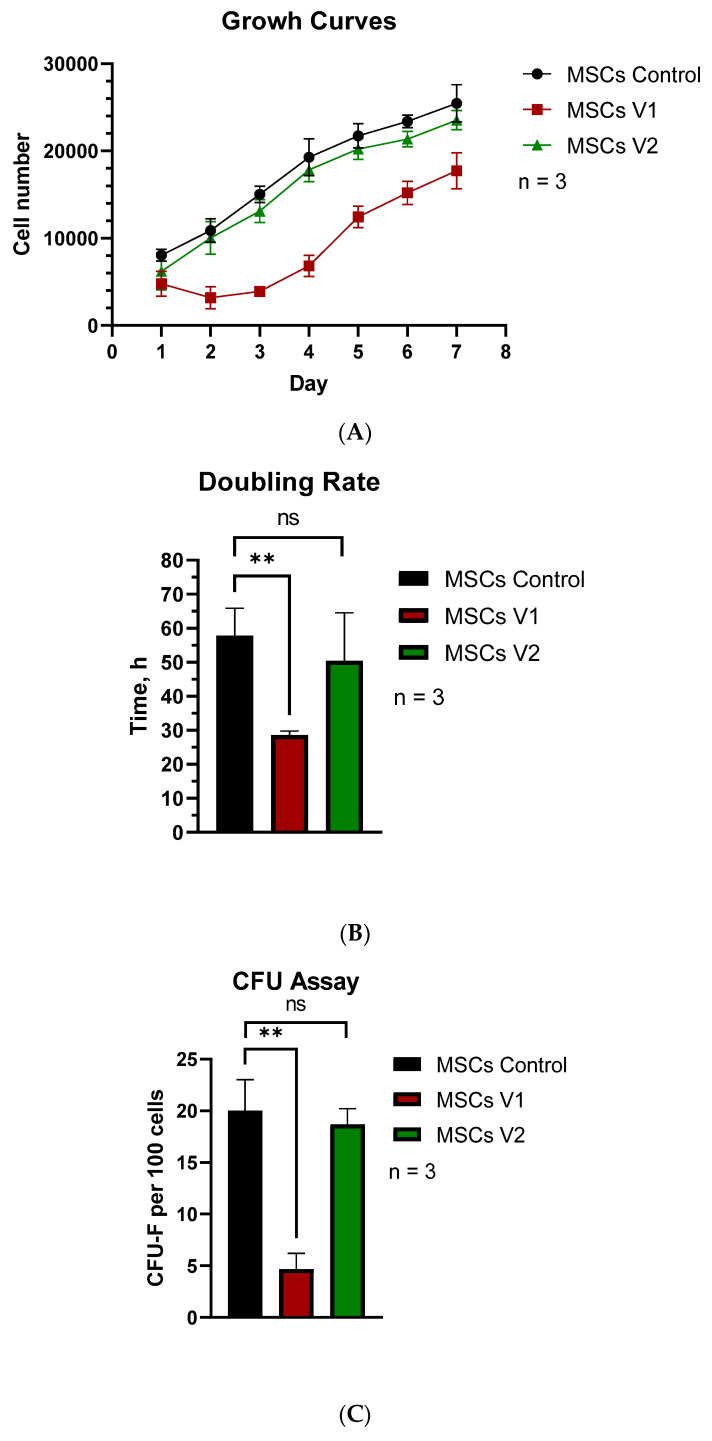
Growth characteristics of MSCs: (**A**) Growth curves of obtained cell lines; (**B**) their doubling rate, *p* = 0.034; (**C**) CFU assay of MSCs, *p* = 0.014. All experiments were performed in n = 3 replicas. Two-way ANOVA was used to perform statistical analysis. ns—not significant, **—*p* < 0.01.

First, growth curves were generated for each obtained cell line (Figure 3A). MSCs.V1 exhibited a distinctly different growth pattern compared to MSCs.Control and MSCs.V2, which behaved similarly. Specifically, MSCs.V1 required approximately three days post-passage to initiate exponential growth, while the other two cell cultures began this phase the day after passage.

Subsequently, we calculated the doubling rate using Parkinson’s formula (Figure 3B). Parallel testing of the cell lines was conducted using a colony-forming unit assay. Cells were seeded sparsely and cultured for a week. The results indicated that MSCs.V1 performed worse than the other cell lines, with no statistically significant difference observed between MSCs.Control and MSCs.V2 (Figure 3C). These data are consistent with results from other performed tests.

### 4.3. Surface Marker Analysis

For the next phase of our analysis, we examined surface markers by flow cytometry. It is well known that MSCs are positive for CD44, CD90, CD105, and CD73 [15], while they must be negative for markers of hematopoietic cell lineage such as CD34, CD11b, CD19, CD45, and HLA-DR [16].

**Figure 4:**
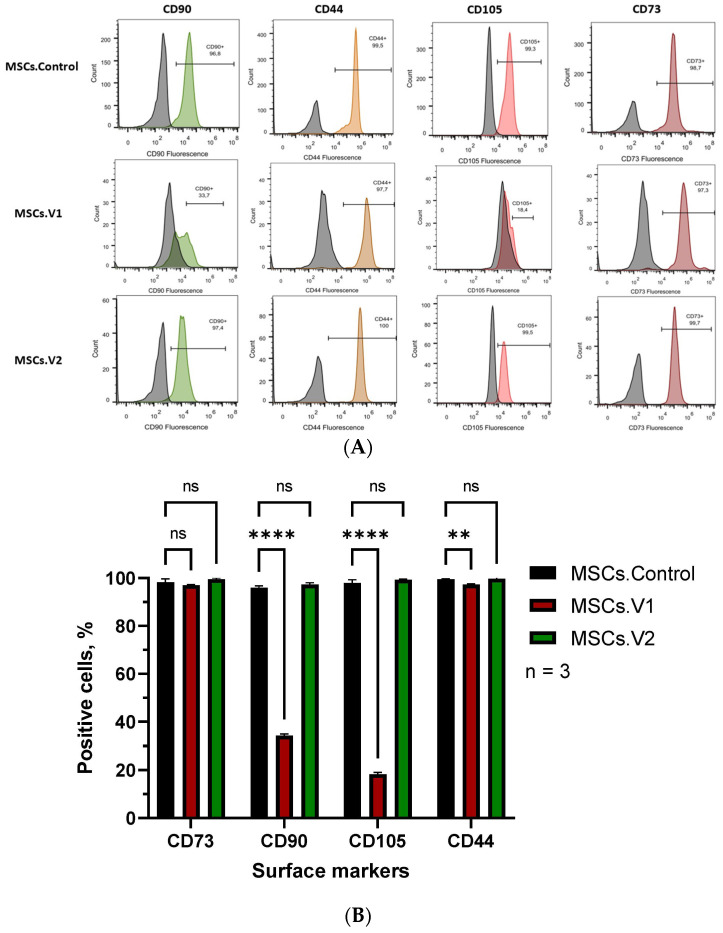

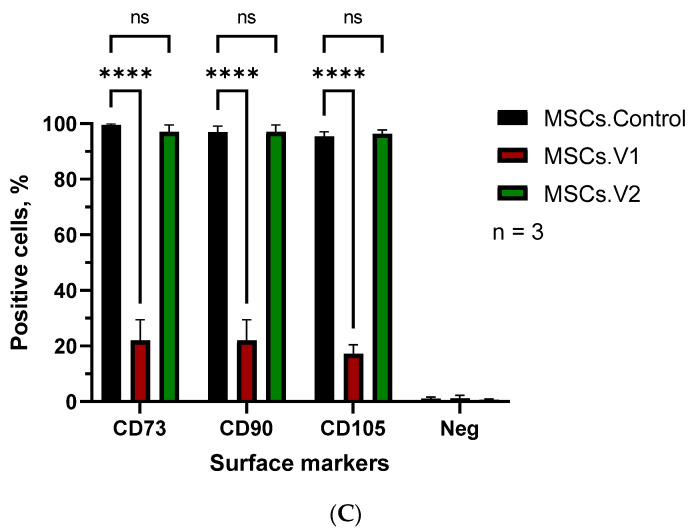
Surface marker analysis: (**A**) Representative images of cell cytometry and (**B**) analysis of staining with one surface marker; (**C**) co-staining of different types of MSCs with CD90-FITC, CD105-PerCP-Cy5.5, CD73-APC, and panel of negative markers (Neg): CD34-PE, CD11b-PE, CD19-PE, CD45-PE, and HLA-DR-PE. All experiments were performed in n = 3 replicas. Two-way ANOVA was used to perform statistical analysis; ns—not significant, **—*p* < 0.01, ****—*p* < 0.0001.

First, we stained cells with single markers: CD90, CD44, CD105, and CD73. The results showed that almost all of the MSCs.Control and MSCs.V2 cell lines expressed these surface markers. Largely, the MSCs.V1 cell line was CD44+ and CD73+, and approximately 35% and 20% were CD90+ and CD105+ (Figure 4A,B). 

Next, we performed multiple staining of all the obtained cell lines. We wanted to know how many cells co-expressed CD73, CD90, and CD105—positive and were CD34, CD11b, CD19, CD45, and HLA-DR—negative. The results showed that both MSCs.Control and MSCs.V2 co-expressed all positive markers and did not express any markers from the negative panel (Neg), without any statistical differences in the expression profile. Notably, only approximately 20% of the MSCs.V1 cell line co-expressed CD73, CD90, and CD105, while neither of the hematopoietic markers were expressed (Figure 4C).

### 4.4. Differentiation Potential

Finally, we decided to check the ability of the obtained cell lines to differentiate into three different lineages: osteogenic, chondrogenic, and adipogenic. All differentiations were performed in the same conditions with three repeats.

**Figure 5:**
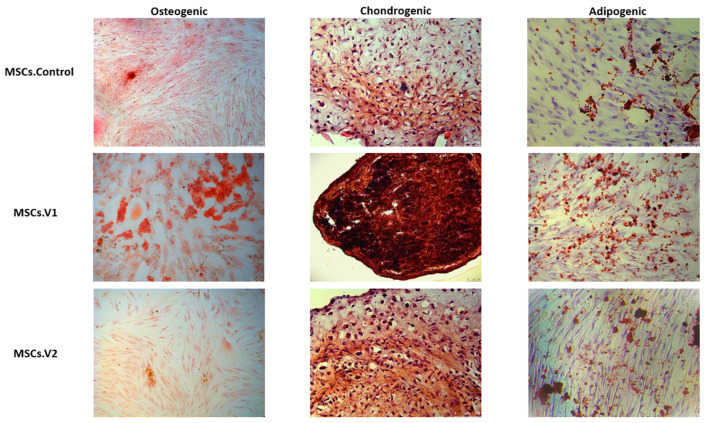
Further differentiation of obtained cell lines to osteogenic lineage (first column, Alizarin Red staining), chondrogenic lineage (second column, Safranin O. staining), and adipogenic lineage (third column, Oil Red O. Staining). Scale bar: 100 mkm.

To identify calcium salts deposited in tissues, Alizarin Red dye was utilized [17]. Osteogenic differentiation revealed that MSCs.V2 had lower potential compared to MSCs.Control, as evidenced by the reduced intensity of red-stained calcium deposits. Despite the monolayer differentiation not being successful in MSCs.V1 cultures and the attached cells forming differentiated clusters, MSCs.V1 exhibited the highest efficiency in osteogenic lineage differentiation (Figure 5).

It is well known that Safranin O. colors glycosaminoglycan into red–orange tones [18]. To validate the effectiveness of chondrogenic differentiation, we employed Safranin O. staining. The MSCs.V2 cell line demonstrated superior chondrogenic differentiation compared to the MSCs.Control cell line, as indicated by the increased red saturation. Notably, MSCs.V1 exhibited the best differentiation in this lineage, despite the 3D chondrogenic culture growing to the smallest size and showing minimal presence of the intercellular matrix (Figure 5).

To identify the presence of adipocytes, we used Oil Red O dye, which binds to neutral triglycerides and lipids [19]. During differentiation into the adipogenic lineage, no visible differences were observed between the MSCs.Control and MSCs.V2 cell lines. MSCs.V1 differentiation performed no worse than other cell cultures (Figure 5). Finally, all cell lines successfully underwent adipogenic differentiation.

## 5. Discussion

In this study, we explored various protocols to derive mesenchymal stem cells (MSCs) from embryonic stem cells (ESCs). Our initial attempts to implement a one-step differentiation protocol using different matrixes and combinations of serum-containing media were unsuccessful. Consequently, we adopted a two-step differentiation strategy, starting with early mesoderm induction. After confirming successful differentiation at the first stage (Figure 1, we proceeded with two new differentiation approaches (MSCs.V1 and MSCs.V2) alongside a positive control (MSCs.Control), using a commercial differentiation kit. At the end of the differentiation process, we observed that the MSCs.Control and MSCs.V2 cell lines exhibited similar morphology and size, whereas MSCs.V1 cells were larger and more heterogeneous (Figure 2).

Next, we evaluated the growth rate and colony-forming ability of the cells post-seeding. Growth curves and doubling time calculations revealed that MSCs.V2 did not differ from MSCs.Control (Figure 3A,B). MSCs.V1 took longer to enter the exponential growth phase (Figure 3A), but three days post-seeding, they began doubling more rapidly than the other cell lines (Figure 3B). Colony-forming assays showed that the MSCs.Control cell line formed the same number of colonies as the MSCs.V2 cell line, while MSCs.V1 almost failed this test (Figure 3C).

Different differentiation protocols yield varying results in different assays. The MSCs.V2 medium produced results comparable to the control and was more user-friendly as it did not require supplementation, hence it is recommended. Serum-containing protocols produced interesting results. The commercial kit demonstrated better cell viability and proliferation compared to “homebrew” protocols, although both parameters were inferior to serum-free media. Additionally, serum-containing media resulted in greater population heterogeneity, both morphologically and in surface marker expression.

Despite the International Society for Cellular Therapy (ISCT) defining human MSCs by the expression of CD73, CD90, and CD105, these markers are expressed on a wide variety of cells [20]. The combination of CD73 and CD44 with the absence of CD105 and CD90 demonstrated by MSCs.V1 cells does not fit the classical MSC profile. These cells may represent fibroblasts, epithelial cells, certain cancer cells, or specific immune cell subpopulations. Further characterization using additional markers and functional assays is necessary to accurately determine the cell type.

The criteria for excluding cells from the MSC population currently rely on hematopoietic stem cell markers, primarily because these markers are well characterized. If markers for stem cell populations from other organs were known, we would likely subdivide the CD73+, CD44+, CD105+, CD90+ population into many subtypes. Despite these complexities, differentiation into the three classical tissue types was successful. The probable reason for such difference in the obtained cell lines may be explained by the composition of the serum-containing media not being clearly defined. It may differ even from batch to batch from one manufacturer. For this reason, it is probable that serum-containing media include numerous inducers of differentiation into various cell types, resulting in a low population of true MSCs in such cultures. However, this approach could be valuable for generating new, poorly described cell types with unknown differentiation processes. For obtaining a pure MSC population, serum-free media should be used.

## 6. Conclusions

In conclusion, our study provides insights into diverse methodologies for generating MSCs from ESCs and highlights the influence of differentiation protocols on cell phenotype and functionality. The findings underscore the need for standardized protocols and comprehensive characterization to ensure the reproducibility and reliability of MSCs derived from PSCs for future therapeutic applications and experimental studies in regenerative medicine.

## Data Availability

The raw data supporting the conclusions of this article will be made available by the authors on request.
